# Heterologous Cortical Lamina vs. Titanium Preformed Mesh Reconstruction in Orbital Fracture: A Retrospective Observational Study

**DOI:** 10.3390/jcm14134668

**Published:** 2025-07-01

**Authors:** Valentino Vellone, Maria Elisa Giovannoni, Antonio Ricciardi, Umberto Committeri, Danilo Alunni Fegatelli, Fabrizio Spallaccia

**Affiliations:** 1 Department of Life Science, Health, and Health Professions, Università degli Studi “Link”, 00165 Rome, Italy; d.alunnifegatelli@unilink.it; 2 Maxillofacial Surgery Unit, University of Siena, Policlinico Santa Maria alle Scotte, Viale Mario Bracci, 53100 Siena, Italy; m.giovannoni4@student.unisi.it (M.E.G.); a.ricciardi5@student.unisi.it (A.R.); 3 Maxillofacial Surgery Unit, “S. Maria” Hospital, 05100 Terni, Italy; umberto.committeri@aospterni.it (U.C.); f.spallaccia@aospterni.it (F.S.)

**Keywords:** orbital fracture, heterologous cortical lamina, titanium mesh, blowout fracture, maxillofacial trauma, orbital reconstruction, postoperative complications

## Abstract

**Background/Objectives**: Orbital fractures are common facial injuries that require precise reconstruction to restore both function and esthetics. Heterologous cortical lamina and titanium preformed meshes are widely used for orbital wall reconstruction; however, comparative data on their outcomes remain limited. **Methods**: This retrospective observational study analyzed 67 patients treated for orbital fractures at Santa Maria Hospital, Terni, between January 2021 and November 2024. Patients underwent orbital reconstruction using either a heterologous cortical lamina or titanium mesh. Clinical data, including demographics, trauma etiology, fracture characteristics, surgical approach, and postoperative complications were collected. Outcomes such as diplopia, enophthalmos, ocular motility, and sensory impairment were assessed preoperatively and postoperatively and compared between groups. Statistical analyses included Chi-square and Mann–Whitney U tests, with logistic regression to identify risk factors for complications. **Results**: Accidental falls were the leading cause of injury (46.3%), with the orbital floor being the most commonly affected site (83.6%). Postoperative complications occurred in 15% of patients, with diplopia significantly reduced from 47.8% preoperatively to 10.4% postoperatively (*p* < 0.05). Sensory impairment and motility restrictions also improved significantly. Patients reconstructed using heterologous cortical lamina experienced significantly fewer postoperative complications compared to those treated with titanium mesh (OR = 0.171, 95% CI: 0.023–0.799, *p* = 0.040). **Conclusions**: Both heterologous cortical lamina and titanium mesh provide effective orbital reconstruction; however, the heterologous cortical lamina was associated with fewer postoperative complications, particularly diplopia and sensory impairment. Material selection should consider the fracture complexity, patient characteristics, and potential long-term outcomes.

## 1. Introduction

Orbits are bony cavities each housing the globe and its associated structures. They are formed by seven bones: zygomatic, sphenoid, maxillary, frontal, lacrimal, palatine, and ethmoid [1].

Orbital fractures are traumatic injuries usually caused by accidents, sports, falls, or assaults. Fractures of the orbit may be seen in different scenarios of direct and indirect trauma to the globe or orbital, facial, or cranial bones. They occur frequently because of blunt orbital and midfacial traumas and may involve ocular injuries.

According to the Cramer et al. classification, orbital fractures have been classified into impure (involving neighboring bones) and pure (limited to orbital walls) [2].

The floor and the medial wall of the orbit are where orbital blowout fractures most frequently occur because they are the thinnest parts [3].

According to many authors, males are more commonly affected than females and most fractures occur in patients under the age of 30 [4,5].

Diplopia, eye movement limitation (due to entrapment of the inferior rectus muscle), hypoesthesia (due to involvement of the infraorbital nerve), and enophthalmos are frequently linked signs and symptoms [6].

An attentive assessment of the orbit injury may include examination of the facial bones, eyelids, and surrounding soft tissue. Furthermore, the inspection of the eye globe, eye movements, and visual acuity need to be carefully evaluated [6].

A definitive diagnosis is confirmed by computed tomography of the orbit. Immediate surgical intervention to restore the anatomic structure of the orbit is therefore essential for improving visual function and orbital appearance [7]. As a result, implants are essential for reestablishing the orbital cavity’s natural and functional anatomic structure.

For orbital wall rebuilding, three different kinds of materials are available and widely explored: autologous, allogenic, and alloplastic [8,9].

The authors selected titanium mesh or heterologous cortical lamina for orbital reconstruction because these are commonly used options in clinical practice. Titanium mesh is a widely accepted and established standard due to its strength and malleability, while heterologous cortical lamina has demonstrated promising results in terms of integration and bone remodeling, particularly in cases requiring biological incorporation with host tissues [10]. Its ability to serve as a scaffold for new bone formation and its reduced risk of infection compared to synthetic materials have been key advantages. Conversely, titanium mesh offers immediate structural stability and has been extensively used in large and irregular defects [11]. However, concerns regarding long-term complications, such as implant exposure, infection, and resorption of surrounding bone, continue to be relevant. Comparing these two materials in terms of surgical feasibility, postoperative outcomes, and long-term durability is essential for optimizing reconstructive strategies.

The aim of this study is to evaluate the pattern and demographic characteristics of patients with orbital wall fractures treated at Santa Maria Hospital in Terni. The relationships between age, sex, etiology, fracture site, repair time, material, and reconstruction technique are analyzed. Furthermore, this study compares the outcomes in patients treated with heterologous cortical lamina versus titanium mesh, testing the hypothesis that heterologous cortical lamina could be associated with a reduced incidence of postoperative complications compared to titanium mesh in orbital fracture reconstruction.

## 2. Materials and Methods

In total, 75 patients with a diagnosis of orbital wall fractures from January 2021 to November 2024 and treated at Santa Maria Hospital (Terni) were admitted to this study.

The hospital’s medical records provided the authors with the information they needed. Sex, age, trauma origin, fracture type and site, anatomical structure involved, symptoms and clinical signs, Hess Lancaster test, type of surgery, and surgical approach to the fracture site were among these details and were used as inclusion criteria. Patients with incomplete records were excluded from this study.

A total of 67 of the 75 initial patients (89%) were included in this study; 8 (11%) of them were not included because they did not fit the criteria.

Patients were grouped into ten-year intervals: under 10 years old (y.o.), 11 to 20 y.o., 21 to 30 y.o., 31 to 40 y.o., 41 to 50 y.o., 51 to 60 y.o., 61 to 70 y.o., 71 to 80 y.o., 81 to 90 y.o., and above 90. Six causes of trauma were considered: road accidents, physical assault, accidental falls, work injuries, sports injuries, and other unspecified causes.

Diagnosis and treatment were based on physical examinations and computed tomography (CT) scans of the orbit in axial, sagittal, and coronal projection. The CT scans helped us also find out if there were other anatomic structures involved such as zygomatic bone, the nose, and maxilla.

Every patient underwent an ophthalmological examination, including the Hess Lancaster test, on the day of admission, before surgery, following surgery once the swelling had subsided, and at 6-month follow-up. Clinical examination for diplopia, enophthalmos, mobility, and sensitivity of the second branch of the trigeminal nerve were performed by the same surgeon who performed the operation (V.V or F.S), before and after surgery.

The scores developed by Jaquiéry et al. [12] were used to categorize the fractures (Table 1).

To accurately determine the healthcare costs for the National Health System, the number of days spent in the hospital before and after surgery was examined.

The existence of symptoms including diplopia, enophthalmos, impaired eye motility, and a fracture bigger than 1 cm in diameter on the CT scan was used to establish the surgical indications.

For orbital reconstruction, four distinct surgical approaches were employed: infraorbital, transconjunctival, subciliar, superciliary, transcaruncolar, and a combined approach.

All operations were conducted by the same maxillofacial surgeons (V.V and F.S). For the orbital floor, medial, and lateral wall reconstruction, the authors used heterologous bone lamina, thickness 0.7 mm (OsteoBiol^®^ Lamina, Tecnoss^®^, Giaveno, Italy), or titanium mesh in large orbital floor defects.

To calculate the cost of healthcare for the National Health System, hospital LOS before and after surgery were considered.

It was on the basis of the presence of symptoms of diplopia and enophthalmos, limitation of eye movement, and the presence of a fragment larger than 1 cm from the base of the CT scan that the indications for surgery were determined.

Orbital reconstruction was performed using four different operative paths: infraorbital, transconjunctival, subciliar—an uncommon option in Europe—superciliary, transcaruncolar, and combined techniques. 

The choice of reconstruction material in orbital floor defects is a complex decision based on defect size and nature as well as surgeon preference and experience. The biomechanical properties, biocompatibility, and complication potential of the material are taken into account. Surgeons reach for materials they think will achieve the best results with the lowest risk of complications.

This study was carried out in accordance with the principles of the Declaration of Helsinki and was approved by the Santa Maria Hospital Ethical Committee (TRAUMAX). All the patients included signed a detailed written informed consent form.

### Statistical Methods

Numerical variables were summarized as the mean (standard deviation, SD) and median (interquartile range, IQR). Categorical variables were expressed as frequencies and percentages. Differences among groups were evaluated using the Mann–Whitney U test and Chi-square test as appropriate.

Logistic regression analysis was performed to evaluate the association between potential risk factors and postoperative complications; results were reported as odds ratios (ORs) with corresponding 95% confidence intervals (CIs).

A *p*-value < 0.05 was considered statistically significant. All analyses were conducted using R software (version 4.4.2).

## 3. Results

This study analyzed a cohort of 67 patients who underwent treatment for orbital fractures, comparing outcomes between those who developed postoperative complications (n = 10, 15%) and those who did not (n = 57, 85%).

Patients without complications had a higher mean age (55.4 year, SD = 19.98) compared to those with complications (45.75 year, SD = 25.09), though this difference was not statistically significant (*p* = 0.173). Sex distribution was similar between the two groups, with females accounting for 36.8% of cases in the no-complications group and 30.0% in the complications group (*p* = 0.953).

Among patients, accidental falls accounted for 46.3% (31 patients) of maxillofacial trauma, followed by physical assaults (13 cases, 19.4%), sports injuries (9 cases, 13.4%), other unspecified causes (6 cases, 8.9%), road accidents (5 cases, 7.5%), and work injuries (3 cases, 4.5%).

Regarding fracture characteristics, the orbital floor was the most affected site (83.6% of cases), with no significant difference between the other groups (*p* = 0.679).

Fracture classification showed a higher proportion of Class III fractures (42.9%) among patients with complications, but the association did not reach statistical significance (*p* = 0.068).

The timing of surgical intervention varied, with most cases undergoing surgery beyond 72 h (34.3%), followed by interventions between 24 and 72 h (32.8%) and within the first 24 h (32.8%) (Table 2).

A higher percentage of patients in the complications group (60%) underwent surgery within the first 24 h, though this finding was not statistically significant (*p* = 0.140).

Fracture type revealed a higher rate of Class III fractures (42.9%) in patients who experienced complications, albeit not achieving statistical significance (*p* = 0.068). Surgical management was initiated after 72 h in most cases (34.3%), followed by surgeries performed between day 2 and 3 (32.8%) and within the first 24 h (32.8%).

The proportion of patients that underwent surgeries in the first 24 h was higher in the complications group compared to the no-complications group (60%), but this difference did not achieve statistical significance (*p* = 0.140).

Preoperative diplopia was found in 47.8% of all tested individuals and preoperative enophthalmos and MOE were present in 1.5% and in 9.0%, respectively. Sensory type dysfunction was observed in 26.9% of patients.

Following surgery, diplopia decreased markedly to 10.4%, and none of the patients developed enophthalmos. Limitation of mobility remained in 1.5% of respondents, and 4.5% of household members had sensory limitations.

Ectropion was also an uncommon complication, presenting at only 1.5% after operation.

Multivariable analysis of the risk factors showed that the use of a bone graft for floor fractures was significantly associated with a lower rate of postoperative complications (OR = 0.171, 95% CI: 0.023–0.799, *p* = 0.040) (Table 3 and Table 4).

## 4. Discussion

The goal of our study was to report our experience with surgical therapy for orbital wall fractures. Our knowledge of the demographics, clinical characteristics, and surgical outcomes of ocular blowout fractures is improved by the current retrospective investigation.

In general, the epidemiological parameters change depending on the study’s period, population demography, culture, and social aspects [13].

Accidental falls were the main cause of maxillofacial injuries, followed by assaults.

Most of them were male patients (43 patients, 64.2%), while only 24 patients were females (35.8%).

In this study, 67 patients’ demographic data, the anatomical location of fractures, and the cause of ocular blowout fractures were examined. The current study’s findings showed that male patients are more likely than female patients to suffer orbital fractures. Men are more likely to suffer from maxillofacial traumas, particularly during the third and fourth decades due to these decades being the most active in people’s lives [14].

Potential factors that increase the likelihood of orbital fractures in men could account for this: the male population may be more involved in road traffic activities, exhibit aggressive behaviors like physical assault, and work in occupations that carry a higher risk of trauma leading to orbital fractures. The mean age of the female patients in this study, as in others [15], was much greater than that of the male patients, despite Choi et al. [16] finding that male patients with blowout fractures were significantly older than female patients. After the sixth decade, maxillofacial injuries are more common in women. Accidental falls are the primary cause of fractures in this age group due to the bone’s increased brittleness following menopause.

The orbital wall is among the most injured components of the maxillofacial skeleton following midfacial trauma. Blowout fractures can result in a variety of functional and cosmetic aftereffects. The materials utilized to bridge the orbital walls have a significant impact on preventing these complications from developing into long-term issues [17].

Even though post-traumatic orbital injuries are common, there is still disagreement in the literature on the best course of treatment for this patient group. The time of surgery (i.e., when post-injury elective orbital reconstruction should occur) as well as the surgical technique and implant type used for orbital architectural reconstruction are all included in this divergence [18].

Numerous implant kinds and materials have been employed, such as autologous and alloplastic implants, as well as resorbable and non-resorbable implants. The literature has described a variety of reconstruction materials for orbital blowout fractures, such as autologous bone transplants (split cranial bone, cartilage, bone fragment, dermal fat, nd rib), allogenic (human dura matter, lyophilized cartilage, banked bone, fascia lata, and heterogenic bovine bone graft), and alloplastic materials (silastic tantalum, stainless steel, titanium, polymethylmethacrylate, polyvinyl sponge, polyurethane, polyethylene, Teflon, hydroxyapatite, gel foam, gel film, and supra mid) [19,20].

Thus, numerous criteria such as fracture site and complexity, operator preference and familiarity, and resource availability affect the choice of implant [21]. The appropriate reconstructive material must possess excellent biocompatibility, ease of manipulation, and robust mechanical strength to sustain the orbital structure. However, there are no widely recognized standards for choosing materials for orbital restoration [22,23]. Our data were compared with the most recent research on postoperative outcomes, which assessed complications and long-lasting symptoms following surgery depending on the materials employed.

There is a substantial correlation between postoperative problems and parameters such as fracture type, surgeon expertise, surgical reconstruction technique, and implant use [24].

Autologous tissues have long been regarded as the accepted method of treating orbital fractures and were the first materials used to rebuild the orbit [25]. Autologous bone (from the patient) has traditionally been used for orbital reconstruction because of its natural strength, rigidity, and resistance to infection. It is harvested from various donor sites such as the skull, ribs, jaw, or hip. However, it requires a second surgical site, increasing surgery time and risk of complications like infection, pain, and scarring. While bone is durable, it is difficult to shape and may break when overly contoured [26]. However, autologous cartilage from the nose, ribs, or ears is another option. It is easier to shape and harvest and causes less donor site damage [27].

Allogenic grafts (from donors) like dura, fascia, or bone avoid the need for a donor site and are readily available. They can help with tissue growth but may resorb faster than autologous materials and carry a small risk of transmitting infections (e.g., HIV, hepatitis, and Creutzfeldt–Jakob disease) [28].

Synthetic (alloplastic) materials are widely used today. They eliminate donor site issues, are easy to use, and come in permanent or resorbable types. They can be divided into permanent and resorbable alloplastic materials.

Titanium and porous polyethylene (MEDPOR) are some examples of permanent alloplastic materials; the first one is strong, easy to shape, and stable over time. It integrates well with bone and is visible on CT scans. However, it may cause soft tissue irritation, infection, or difficulty in removal if complications arise [29]. Meanwhile, porous polyethylene encourages tissue integration, reducing rejection and extrusion. It is soft and easy to handle but not easily seen on CT scans. Still, it is considered safe and effective [30].

On the other hand, resorbable alloplastic materials include the following: Polyglycolic acid, which absorbs within 9 months and works well for small defects but is not suitable for large defects due to weaker support [31]. Vicryl mesh, which is easy to shape but flimsy, requiring multiple layers, and can cause mild inflammation [32]. Polydioxanone, which is suitable for moderate defects. Some studies show mixed results, with some complications like enophthalmos and implant movement, so it is best avoided for large defects [33]. Poly-L/D-Lactic acid, which provides strong support during healing and is fully resorbed, leaving behind new tissue. It has a low complication rate and is effective for large defects, making it a reliable and safe option [34].

In our study, we considered the post-surgery complications to evaluate the differences between titanium mesh and bone graft. We experimented with more complications such as diplopia and sensory impairment in patients who underwent reconstructive orbital floor surgery with titanium mesh.

Titanium provides solid orbital support, has a high osseointegration rate, and has a low resorption potential, but it can also result in a fibrotic reaction. According to the literature, residual diplopia has been noted after its usage; however, problems like enophthalmos and variations in pupillary height are rarely recorded. Furthermore, issues including implant malpositioning, iatrogenic fractures, and screw loss have been brought to light because titanium is a stiff material that needs to be secured with screws [35,36]. According to a retrospective analysis of the literature, orbital adherence syndrome was discovered in 6% of orbital reconstruction procedures with orbital mesh plates [37].

Also other studies have reported this kind of complication with titanium mesh. In these instances, the titanium mesh implant and the orbital contents had experienced a severe fibrotic reaction that determined postoperative diplopia and impairment of eye movements [19].

This study presents several limitations that warrant consideration. First, the retrospective design is inherently susceptible to selection and information biases, which may influence data accuracy and internal validity. Second, although the sample size is sufficient for preliminary comparisons, it remains relatively limited, potentially impacting the statistical power and the external generalizability of the findings. Third, the evaluation of postoperative complications—such as diplopia and sensory disturbances—was conducted by the operating surgeons without blinded assessment, which introduces the risk of observer bias. Moreover, the follow-up period, while adequate for identifying early postoperative complications, may be insufficient to capture long-term outcomes or delayed adverse events. To substantiate these results, future prospective, multicenter investigations with larger cohorts, standardized protocols, blinded outcome assessments, and extended follow-up are recommended.

## 5. Conclusions

This retrospective observational study highlights that both heterologous cortical lamina and titanium mesh are effective and reliable options for orbital wall reconstruction following traumatic fractures. However, the findings of this study suggest that the use of heterologous cortical lamina is associated with a reduced incidence of postoperative complications, particularly regarding diplopia and sensory impairment, compared to titanium mesh. These results support the evidence in favor of the use of biologically active materials in orbital reconstruction, particularly in cases where minimizing long-term morbidity is important. However, the selection of reconstructive material should be customized to the patient’s fracture pattern, clinical presentation, and surgeon expertise. It is recommended that further prospective, randomized studies are conducted, with larger cohorts and long-term follow-up to validate these findings and establish standardized guidelines for material selection in orbital wall reconstruction.

## Figures and Tables

**Table 1:** Classification of orbital wall defects according to Jaquiéry et al. [12].

Class	Description
I	Isolated defect of the orbital floor or the medial wall, 1–2 cm^2^, in the anterior two-thirds
II	Defect of the orbital floor or medial wall, >2 cm^2^, in the anterior two-thirds Bony ledge preserved at the medial margin of the infraorbital fissure
III	Defect of the orbital floor or medial wall, >2 cm^2^, in the anterior two-thirds Missing bony ledge medial to the infraorbital fissure
IV	Defect of the entire orbital floor and the medial wall, extending into the posterior third
V	Same as Class IV, defect extending into the orbital roof

**Table 2:** Demographic and clinical/surgical characteristics of orbital fracture patients. Comparison between patients without (n = 57) and with (n = 10) postoperative complications.

Variable	All (n = 67)	No Complications (n = 57)	Complications (n = 10)	*p*-Value
**Age (mean ± SD)**	53.4 (21.0)	55 (20.0)	44.2 (25.3)	0.173
**Age (median [IQR])**	56 (34–70)	57 (39–71)	40 (21–66)	
**Sex (Female)**	24 (35.8%)	21 (36.8%)	3 (30.0%)	0.953
**Fracture Site**				0.679
Floor	56 (83.6%)	48 (84.2%)	8 (80.0%)	
Medial wall	1 (1.5%)	1 (1.8%)	0 (0.0%)	
Floor + Medial wall	7 (10.4%)	6 (10.5%)	1 (10.0%)	
Floor + Medial + Lateral walls	1 (1.5%)	1 (1.8%)	0 (0.0%)	
Floor + Lateral wall	2 (3.0%)	1 (1.8%)	1 (10.0%)	
**Fracture Classification**				0.068
Class I	6 (12.2%)	6 (14.3%)	0 (0.0%)	
Class II	28 (57.1%)	25 (59.5%)	3 (42.9%)	
Class III	12 (24.5%)	9 (21.4%)	3 (42.9%)	
Class IV	2 (4.1%)	2 (4.8%)	0 (0.0%)	
Class V	1 (2.0%)	0 (0.0%)	1 (14.3%)	
**Timing of Surgery**				0.140
0–24 h	22 (32.8%)	16 (28.1%)	6 (60.0%)	
24–72 h	22 (32.8%)	20 (35.1%)	2 (20.0%)	
>72 h	23 (34.3%)	21 (36.8%)	2 (20.0%)	

**Table 3:** Logistic regression—risk factors for postoperative complications in orbital fractures.

Variable	OR	95% CI	*p*-Value
Preoperative complications	8.596	1.400–167.345	0.052
Treatment with bone graft (floor)	0.171	0.023–0.799	0.040

**Table 4:** Additional pre- and postoperative symptom frequencies.

Symptom	Preoperative (n = 67)	Postoperative (n = 67)
Diplopia	32 (47.8%)	7 (10.4%)
Enophthalmos	1 (1.5%)	0 (0.0%)
Motility restriction (MOE)	6 (9.0%)	1 (1.5%)
Sensory impairment	18 (26.9%)	3 (4.5%)
Ectropion	-	1 (1.5%)

## Data Availability

The original contributions presented in this study are included in the article. Further inquiries can be directed to the corresponding author.
